# Factors associated with eHealth literacy among people with systemic sclerosis: A Scleroderma Patient-centred Intervention Network (SPIN) Cohort cross-sectional study

**DOI:** 10.1177/23971983251376428

**Published:** 2025-09-29

**Authors:** Natalie Co, Claire Adams, Marie-Eve Carrier, Meira Golberg, Elsa-Lynn Nassar, Linda Kwakkenbos, Susan J Bartlett, Catherine Fortuné, Amy Gietzen, Geneviève Guillot, Amanda Lawrie-Jones, Vanessa L Malcarne, Maureen D Mayes, Michelle Richard, Luc Mouthon, Andrea Benedetti, Brett D Thombs

**Affiliations:** 1 Lady Davis Institute for Medical Research, Jewish General Hospital, Montreal, QC, Canada; 2 Department of Psychology, McGill University, Montreal, QC, Canada; 3 Department of Psychiatry, McGill University, Montreal, QC, Canada; 4 Department of Clinical Psychology, Behavioural Science Institute, Radboud University, Nijmegen, The Netherlands; 5 Department of IQ Health, Radboud University Medical Center, Nijmegen, The Netherlands; 6 Radboudumc Center for Mindfulness, Department of Psychiatry, Radboud University Medical Center, Nijmegen, The Netherlands; 7 Department of Medicine, McGill University, Montreal, QC, Canada; 8 Research Institute of the McGill University Health Centre, Montreal, QC, Canada; 9 Ottawa Scleroderma Support Group, Ottawa, ON, Canada; 10 Steffens Scleroderma Foundation, Albany, NY, USA; 11 Sclérodermie Québec, Longueuil, QC, Canada; 12 Scleroderma Australia, Melbourne, VIC, Australia; 13 Scleroderma Victoria, Melbourne, VIC, Australia; 14 Department of Psychology, San Diego State University, San Diego, CA, USA; 15 San Diego State University/University of California, San Diego Joint Doctoral Program in Clinical Psychology, San Diego, CA, USA; 16 University of Texas McGovern School of Medicine, Houston, TX, USA; 17 Scleroderma Atlantic, Halifax, NS, Canada; 18 Service de Médecine Interne, Centre de Référence Maladies Autoimmunes et Autoinflammatoires Systémiques Rares d’Ile de France, de l’Est et de l’Ouest, Hôpital Cochin, Paris, France; 19 Assistance Publique Hôpitaux de Paris-Centre, Hôpital Cochin, Université Paris Cité, Paris, France; 20 Department of Epidemiology, Biostatistics, and Occupational Health, McGill University, Montreal, QC, Canada; 21 Respiratory Epidemiology and Clinical Research Unit, McGill University Health Centre, Montreal, QC, Canada

**Keywords:** Cross-sectional study, eHealth literacy, health literacy, systemic sclerosis

## Abstract

**Introduction/objective:**

eHealth literacy reflects the ability to obtain, understand, and evaluate health information from electronic sources and apply this information to health problems. Our objective was to evaluate sociodemographic (age, sex, race or ethnicity, education, marital status, country, residence location) and disease factors (duration, subtype) associations with eHealth literacy among individuals with systemic sclerosis (SSc).

**Methods:**

Scleroderma Patient-centred Intervention Network (SPIN) Cohort participants completed the 8-item eHealth Literacy Scale (eHEALS) from January 17 to February 18, 2025. Multivariable linear regression was used to assess associations of sociodemographic and disease characteristics with eHealth literacy.

**Results:**

The 333 participants were from France (N = 116, 35%), Canada (N = 90, 27%), the United States (N = 85, 26%), the United Kingdom (N = 32, 10%), and Australia, Mexico, or Spain (N = 10, 3%). Most participants were female (N = 295, 89%), White (N = 268, 80%), and had limited SSc (N = 206, 62%). Compared to the United States, participants from Canada (−2.2 points, 95% CI −4.2 to −0.1; standardized mean difference (SMD) = –0.33) and France (−4.2 points, 95% CI −6.2 to −2.3; SMD = −0.64) had significantly lower eHEALS scores. Age, sex, race or ethnicity, marital status, education level, time since first non-Raynaud’s symptom onset, and disease subtype were not associated with eHEALS scores.

**Conclusion:**

eHealth literacy in SSc was not associated with age and education level as in some other studies but was associated with country. Future research should examine country-level differences in eHealth literacy for individuals with SSc.

## Introduction

Health literacy reflects an individual’s ability to find, understand, evaluate, and use information to make decisions about their health.^1,2^ Models of health literacy conceptualize this construct as a set of knowledge and skills, or a hierarchy of functions, related to (1) having knowledge of health, healthcare, and health systems; (2) processing and using information in various formats in relation to health and healthcare; and (3) maintaining health through self-management and working in partnerships with healthcare providers.^3^ The concept of eHealth literacy was subsequently established to encompass health information obtained from electronic sources, including media literacy and computer literacy, and traditional literacy and numeracy.^4^ eHealth literacy reflects the ability to obtain, understand, and evaluate electronic health information and apply this information to inform understandings about health and healthcare.^4^

More concretely, greater health literacy and eHealth literacy enable individuals to better maintain or improve their health by more effectively working in partnership with their healthcare providers as active, knowledgeable participants in their care. For instance, health literacy supports shared decision-making, a process in which patients play an active role in maintaining or improving their health by participating in a two-way relationship with their healthcare provider and selecting care options in light of both their values and preferences and physician knowledge and recommendations.^5^ Health literacy and shared decision-making have been associated with outcomes that include positive quality of care indicators, better patient-reported health outcomes, and reductions in emergency department visits.^6,7^

Given the role health literacy plays in positive patient outcomes, it is important to understand what factors are associated with health literacy. Among large general population studies, a nationwide study in the United States (N = 14,592)^8^ and a multinational study of 8 European countries (total N = 8,000)^9^ reported that younger age, higher socioeconomic status, and higher education level were associated with greater health literacy. Systematic reviews and large-scale epidemiological studies have found that greater health literacy among people with chronic diseases is associated with female sex, higher socioeconomic status, and higher educational level.^10–15^ A 2023 systematic review and meta-analysis found similar predictors for eHealth literacy.^16^ Race or ethnicity may be associated with health and eHealth literacy,^10,14,17^ although categorization of race or ethnicity and its meaning in terms of socioeconomic positioning is often context- or country-specific.

Beyond these sociodemographic characteristics, it may be especially important to examine health and eHealth literacy in the context of rare diseases. Barriers to health and ehealth literacy in rare diseases may include the complexity of managing and treating some conditions and the lack of patient-oriented information sources, making it particularly difficult to find information and decide if it applies to them.^18^ Even then, there is only limited research on health literacy in rare diseases, including among people with systemic sclerosis (SSc; scleroderma), restricting the availability of evidence-based information. SSc is a rare autoimmune disease involving microvascular damage and characterized by fibrosis of the skin and internal organs, including the lungs, gastrointestinal tract, heart and kidneys.^19^ SSc onset typically occurs around 50 years, and about 85% of people with SSc are female.^20,21^ Global prevalence may be 15 to 20 per 100,000 people,^22^ although a 2024 Quebec population-based study estimated 29 per 100,000 (48 female, 10 male).^21^ Mobility limitations, including severely reduced hand function, fatigue, gastrointestinal symptoms, pain, and impaired mental health, are common in patients with SSc.^23–35^

We identified only three studies on health literacy or eHealth literacy in SSc. A survey of 101 people with SSc from Switzerland used study-specific items on eHealth literacy and found that patients with less education reported a lower understanding of health-related information from the Internet and that older age may also be associated with a lower level of understanding.^36^ A study from China developed an SSc-specific health literacy measurement tool,^37^ but the constructs used in the tool deviated from commonly used theoretical frameworks^1,3^ and included treatment compliance, for instance, which is not part of health literacy.^37,38^ The third study, which was conducted in Italy, included 57 participants and provided only descriptive analyses of participants’ sociodemographic and clinical characteristics, responses to items on a health literacy measure, and health literacy levels but did not assess factors associated with health literacy.^39^

To our knowledge, no studies have evaluated factors associated with health literacy or eHealth literacy, as conceptualized in standard health literacy frameworks and with a validated measure,^1,3,4^ among people with SSc. Our objective was to evaluate sociodemographic and disease factors associated with eHealth literacy among individuals with SSc.

## Methods

This was a cross-sectional study that evaluated data from the Scleroderma Patient-centred Intervention Network (SPIN) Cohort.^40–42^ Because methods of studies that use SPIN Cohort data are similar, we adopted some of the same methods as in previous studies. We followed guidance from the Text Recycling Research Project.^43^ We reported results following guidance in the Strengthening the Reporting of Observational Studies in Epidemiology Statement.^44^

### Participants and procedures

The SPIN Cohort is a convenience sample of participants from 7 countries, including Australia, Canada, France, Mexico, Spain, the United Kingdom, and the United States.^40–42^ Eligible participants are recruited by site attending physicians or nurse coordinators during regular outpatient visits. Participants included in the SPIN Cohort must be ⩾18 years of age; fluent in English, French or Spanish; and diagnosed with SSc based on the 2013 ACR/EULAR classification criteria,^45^ as confirmed by a SPIN site physician. After obtaining written informed consent from eligible participants, onsite staff submit an online medical data form and participants receive an automated email with instructions on how to activate their online SPIN account and complete their required measures. SPIN Cohort participants complete outcome measures via an online portal upon enrolment and subsequently every 3 months. The SPIN Cohort study was approved by the Research Ethics Committee of the Centre intégré universitaire de santé et de services sociaux du Centre-Ouest-de-l’Île-de-Montréal (#MP-05-2013-150) and by the ethics committees of all recruiting sites. Participant recruitment is ongoing. This study included data from participants who completed the eHealth Literacy Scale (eHEALS),^46^ which was administered in the SPIN Cohort from January 17 to February 18, 2025.

### Measures

SPIN Cohort participants provide sociodemographic data (race or ethnicity, gender, years of education, relationship status, country, housing location) and complete patient-reported outcomes at baseline and subsequent assessments. At enrolment, physicians report on participants’ age, sex, years since initial onset of non-Raynaud phenomenon symptoms, and SSc subtype (limited, diffuse, sine).

#### eHealth literacy

The eHEALS is an 8-item self-report measure of eHealth literacy that was initially developed in a youth population (N = 664)^46^ and has since been validated in multiple populations, including older adults (N = 866).^47^ The eHEALS captures perceived skills and comfort with obtaining, understanding, and using health information from electronic sources. The eHEALS measures traditional literacy, health literacy, information literacy, scientific literacy, media literacy, and computer literacy, however, the eHEALS does not directly measure required cognitive and educational skills (e.g. numerical literacy, reading skills), as this would require a detailed professional assessment with substantial resource demands.^46^ eHEALS item response options include 1 = strongly disagree, 2 = disagree, 3 = undecided, 4 = agree, 5 = strongly agree (possible score range 8–40). Higher scores indicate better eHealth literacy.^46^ The eHEALS has demonstrated good or high internal consistency and validity across diverse populations,^47,48^ including those with chronic diseases.^49^

### Statistical analysis

We computed descriptive statistics for all variables. Simple linear regression was used to assess unadjusted associations between sociodemographic and medical variables and eHealth literacy. Multivariable linear regression was used to assess the independent association of each variable with eHealth literacy. We identified items to be included in the model *a priori* based on previous studies of factors associated with health literacy in general and major disease populations.^8–15^ Variables included in the main analysis were age (years); male sex (reference = female); non-White (reference = White); education (years); single, divorced, separated, or widowed (reference = married or living as married); Canada, United Kingdom, France, other country (Mexico, Spain, Australia; reference = United States); village or town, suburb, rural, other location (reference = city); time since first non-Raynaud’s symptom (years); diffuse subtype (reference = limited or sine). In addition to regression coefficients, we presented estimates of standardized mean differences (SMD) that represent standard deviations of change, which are typically interpreted as small (0.20), medium (0.50), or large (0.80) effects.^50^

We accounted for missing data by using multiple imputation via chained equations, using the mice package in R.^22^ We generated 20 imputed datasets, using 15 cycles per dataset. Variables included in the mice procedure included all variables in the main regression model, including the outcome variable, and additional disease and sociodemographic variables measured in the SPIN Cohort: age, sex, gender, race or ethnicity, education, marital status, country, location, time since first non-Raynaud’s symptom, disease subtype, smoking status, alcohol consumption, body mass index, and the physical function, anxiety, depression, pain intensity, pain interference, fatigue, sleep, and satisfaction with social roles and activities function domain scores from the PROMIS-29v2.0.^51^ We additionally conducted a complete data analysis that included only participants without any missing variables.

We reported unstandardized regression coefficients with 95% confidence intervals (CIs) and total explained variance for each model (adjusted *R*^2^). All regression analyses were conducted in R (R version 3.6.3, RStudio Version 1.2.5042). For country-level analyses, the United States was chosen as the reference group as it had the largest sample by country and has served as a reference group in previous SPIN studies.

In addition, we observed a strong association between eHealth literacy and country, especially in France, while unexpectedly finding no link to education. Thus, post hoc, we examined bivariate associations of education and eHealth by country and evaluated our multivariable model with all countries except France. We did not attempt to evaluate interactions because of the relatively small number of participants per country.

## Results

Our sample consisted of 333 participants from 46 sites. Participants were predominantly female (N = 295; 89%) and White (N = 268; 80%). The mean (standard deviation (SD)) age was 61.3 (12.0) years, and the mean (SD) education level was 15.7 (4.9) years. Most participants were married or living as married (233; 70%); from France (N = 116; 35%), Canada (N = 90; 27%), or the United States (N = 85; 26%); and lived in cities (N = 119; 36%), suburbs (N = 94; 28%), or villages or towns (N = 80; 24%).

The mean (SD) number of years since first non-Raynaud’s symptoms was 16.7 (9.9), and most participants had limited SSc (N = 206; 62%). Table 1 shows participant sociodemographic and disease characteristics. The mean (SD) eHEALS score was 27.8 (6.6), including 28.0 (6.7) for Canada, 30.1 (5.4) for the United States, 27.8 (6.1) for the United Kingdom, and 26.1 (7.0) for France.

**Table 1.** Sample sociodemographic and disease characteristics in 333 people with systemic sclerosis.

	Mean (SD) or N (%)
Age (years)	61.3 (12.0)
Female sex^a^	295 (88.6%)
Race or ethnicity^b^	
White	268 (80.5%)
Non-White	65 (19.5%)
Education (years)^c^	15.7 (4.9)
Marital status^c^	
Married or living as married	233 (70.0%)
Single	43 (12.9%)
Divorced/separated	43 (12.9%)
Widowed	13 (3.9%)
Country	
Canada	90 (27.0%)
France	116 (34.8%)
United Kingdom	32 (9.6%)
United States	85 (25.5%)
Australia, Mexico, or Spain^d^	10 (3.0%)
Location^e^	
City	119 (35.7%)
Village or town	80 (24.0%)
Suburb	94 (28.2%)
Rural	37 (11.1%)
Time since first non-Raynaud’s symptom (years)^f^	16.7 (9.9)
Diffuse SSc^g^	127 (38.1%)

N: number; SD: standard deviation; SSc: systemic sclerosis.

a Participants also reported gender (cisgender woman, cisgender man, transgender woman, transgender man, non-binary, other). All answered cisgender woman or cisgender man, except 4 participants who either checked more than one option or none.

b Race or ethnicity data are self-reported in each country using standard categories used in each country. Therefore, categories differed between countries.

c N = 332.

d N = 3 from Australia, N = 3 from Mexico, N = 4 from Spain.

e N = 330. 3 open-ended responses were classified into existing categories.

f N = 311.

g Versus limited SSc (N = 196) or sine scleroderma (N = 10).

In the main multivariable analysis (Table 2), age (-0.5 points per 10 years, 95% CI −1.1 to 0.1; SMD = −0.08 per 10 years) and education level (−0.05 points per 5 years; 95% CI −0.8 to 0.7; SMD = −0.01 per 5 years) were not significantly associated with eHEALS scores. Among countries, Canada (−2.2 points, 95% CI −4.2 to −0.1; SMD = −0.33), the United Kingdom (−2.2 points, 95% CI −5.0 to 0.7; SMD = −0.32), and France (−4.2 points, 95% CI −6.2 to −2.3; SMD = −0.64) were associated with lower eHEALS scores compared to the United States, with Canada and France and Canada statistically significantly different. Mexico, Spain, and Australia (−5.8 points, 95% CI −10.1 to −1.4; SMD = −0.87) were also associated with lower scores. Additional variables not statistically significantly associated with eHEALS scores included sex, race or ethnicity, marital status, location, time since first non-Raynaud’s symptom onset, and diffuse subtype. Adjusted R2 for the final model was 0.03.

**Table 2.** Linear regression analysis of sociodemographic and disease characteristic associations with eHealth literacy (N = 333).

	Unadjusted regression coefficient (95% CI)	Adjusted regression coefficient (95% CI)
Sociodemographic variables		
Age (per 10 years)	−0.40 (−1.00, 0.20)	−0.50 (−1.10, 0.10)
Sex (reference = female)	−1.49 (−3.74, 0.76)	−1.38 (−3.67, 0.92)
Non-White (reference = White)	0.44 (−1.37, 2.25)	0.28 (−1.60, 2.17)
Education (per 5 years)	0.15 (−0.60, 0.85)	−0.05 (−0.80, 0.70)
Single, divorced/separated, or widowed (reference = married or living as married)	0.42 (−1.14, 1.99)	0.13 (−1.47, 1.73)
Country (reference = United States)		
Canada	**−2.08 (−4.01, −0.15)**	**−2.16 (−4.18, −0.14)**
United Kingdom	−2.23 (−4.88, 0.41)	−2.15 (−5.03, 0.74)
France	**−3.87 (−5.70, −2.05)**	**−4.23 (−6.21, −2.26)**
Australia, Mexico, or Spain	**−5.05 (−9.31, −0.78)**	**−5.77 (−10.13, −1.40)**
Location (reference = city)		
Village or town	−0.85 (−2.74, 1.05)	−0.88 (−2.89, 1.13)
Suburb	0.35 (−1.46, 2.17)	−0.66 (−2.55, 1.22)
Rural	−0.12 (−2.59, 2.34)	−0.29 (−2.75, 2.18)
Years since first non-Raynaud’s symptom (years)	0.00 (−0.07, 0.08)	−0.01 (−0.09, 0.06)
Diffuse subtype (reference = limited or sine)	0.28 (−1.19, 1.76)	−0.25 (−1.76, 1.27)

CI = confidence interval. Adjusted R^2^ = 0.03. Statistically significant coefficients are shown in bold.

Complete case analysis results, which included 306 participants, were similar to results from the main analysis (see Supplemental Appendix 2).

To better understand the associations between eHealth literacy, country and education, we conducted post hoc analyses. Bivariate analyses by country (Supplemental Appendix 3) found that in Canada, the United Kingdom, and the United States, there were non-statistically significant positive associations of 1.00 to 1.45 points per 5 years with education, however, the estimated association for France was close to zero. Thus, we conducted a multivariable analysis that included all countries except France and found a positive but non-statistically significant association of 1.10 points per 5 years of education (95% CI -0.20 to 2.40; SMD = 0.17 per 5 years). All other model coefficients were similar to those in the main analysis (see Supplemental Appendix 4).

## Discussion

Our main findings were that there were differences between countries in eHealth literacy scores, but the variables that we expected to be associated with eHealth literacy, age and education, were not statistically significantly associated. Participants from Canada, the United Kingdom, and France had lower mean ehealth literacy scores than participants from the United States in our multivariable analysis, although differences were not statistically significant for the United Kingdom. There were only 10 participants from Mexico, Spain, or Australia. The difference between the mean scores of participants from Canada and those from the United States, was of a magnitude that is typically considered a small effect, however, the difference between the mean scores of participants from France and the United States, is of a magnitude typically considered a medium to large effect, meaning participants from France had substantially lower eHealth literacy^51^ eHEALS scores were lower with age, which is the direction of association we expected, although small and not statistically significant. The association with education level was approximately zero. However, when the association with education was examined by country, there were non-statistically significant positive associations for Canada, the United States, and the United Kingdom. The association for France was near zero. Consistent with this, a post hoc multivariable analysis with all countries except France found a positive though statistically insignificant association with education.

To our knowledge, this is the first study that has examined eHealth literacy in an SSc population using the eHEALS measure. The mean (SD) eHEALS score was approximately 28 (7), which is similar to the general Canadian population (N = 10,130, mean = 27, SD not reported)^52^ and individuals from Australia with moderate to high cardiovascular risk in a randomized controlled trial of a consumer-focused e-health strategy (N = 397, mean = 27, SD = 7).^48^ It was lower than older adults from the United States in an online bone health intervention study (N = 866, mean = 31, SD = 6)^47^ and chronic disease patients from the United States recruited online (N = 648, mean = 30, SD = 5).^49^

The most recent meta-analysis on sociodemographic predictors of eHealth literacy^16^ included 36 studies (all used eHEALS) and reported that age was associated with a decrease of 0.05 (16 studies; 95% CI 0.04 to 0.06) eHEALS points per year, which is equivalent to about 0.50 points per 10 years, the same magnitude decrease we found. The authors did not meta-analyse results on education levels but reported that 14 of 27 studies that analysed education a significant positive association between education and eHealth literacy (13 not statistically significant). There were, however, some important differences between studies included in the meta-analysis and our study. Of the 36 studies included, 14 were conducted in Asian countries – including Vietnam, Hong Kong, South Korea, Malaysia, Taiwan, Pakistan, and China – and 3 of the studies were conducted in Ghana and Ethiopia, whereas almost all our data were from North American or Western European countries. In addition, 17 of the studies in the meta-analysis were conducted at clinical sites, whereas ours was administered online in a group of people with SSc who regularly complete study forms via the Internet, some of whom also participate in Internet-based interventions.^53–56^ Our results suggest that, in Canada, the United Kingdom, and the United States, education may be similarly associated with eHealth literacy. This does not appear to be the case in France, where overall eHealth literacy was the lowest.

As shown by the lower scores in France, it is likely cultural differences exist in eHealth literacy between countries. However, to the best of our knowledge, no studies have evaluated factors associated with eHealth literacy in France. Consistent with our findings, though, a study of telehealth use in SSc (N = 314) found that only 35% of participants from France received some or all of their care via telehealth during the pandemic compared to 89% to 96% of participants from Canada, the United Kingdom, and the United States; 73% of participants from France preferred to receive all of their care post-pandemic in person compared to 33% to 43% in the other countries.^57^ Although evidence regarding telehealth use in France is scarce, SPIN physician collaborators from France, including co-authors of this manuscript, report that they predominantly use in-person care because they believe it is necessary to provide quality care. This is consistent with French participant preferences in the study described above.^57^ It is possible that in France, eHealth is rarely used and so eHealth literacy is much less developed across the population, regardless of education levels, due to these attitudinal differences towards eHealth and eHealth care use. The eHEALS measures perceived knowledge, skills, and comfort using electronic resources to make decisions about health and healthcare. It does not measure whether people have the cognitive or educational skills necessary to become eHealth literate or to use eHealth resources in their own healthcare.

In SSc, the Swiss study of eHealth literacy (N = 101)^36^ found that less education was associated with a lower understanding of health-related information based on a single item and that age may have been associated. The study did not, however, report on results of associations with other items, and the single items constructed by the researchers make comparisons difficult. To our knowledge, our study was the first study of eHealth literacy in SSc using the eHEALS, which may be the most commonly used tool for this purpose.^16^ Due to barriers that individuals with rare diseases face in obtaining high-quality, easy-to-understand health information on their condition, it is important to understand what factors may affect eHealth literacy in SSc. This knowledge would help inform clinical practice and the development of eHealth literacy interventions and resources. Future research might further investigate country-level differences in eHEALS scores and in how eHealth resources are used in different health systems.

Strengths of our study include our international sample of participants from 46 SPIN sites across 7 countries, the use of a validated measure of eHealth literacy, and the involvement of people with lived SSc experience via membership on SPIN’s Steering Committee. There are also limitations to consider. First, we collected data via online questionnaires, which could have resulted in higher eHEALS scores since we did not include people who were unable to use digital tools.^16^ This is consistent with the relatively high mean eHEALS score in the SPIN cohort. Moreover, while the eHEALS is a widely used tool, it has not been validated in an SSc population, and therefore the measurement properties of this scale among people with SSc have not been established. In addition, categories for interpretation of eHEALS score levels have not been established. Second, our study was cross-sectional, which did not allow us to infer causality based on our results. Third, the adjusted R^2^ value for our eHealth literacy model was low. High R^2^ values are important in predictive modelling, but much less so when models are used for testing hypotheses about the possible effects of variables of interest. In such models, including models used in the present study, having a sufficiently large sample size to generate reasonably precise parameter estimates is a more important consideration.^58^ A larger sample size would have improved the precision of our model estimates, but we were limited to using data collected in the SPIN Cohort in the period when the eHEALS was administered. Nonetheless, our sample size was much larger than previous studies in SSc, which in this case is a more important consideration than high R^2^ values for hypothesis testing.^58^ Future research could identify other predictors of ehealth literacy that are missing from the model, such as psychosocial or systemic factors, to better explain the mechanisms that underpin ehealth literacy scores.

In summary, we found that age and education level were not significantly associated with eHealth literacy among people with SSc, although it is possible that this may be country specific. People with SSc from France had substantially lower eHealth literacy compared to those living in the United States, which was our reference country. When data from countries other than France were analysed, it appeared that education may be associated with eHealth literacy, as expected, although this was statistically insignificant. More studies are needed to better understand factors associated with eHealth literacy in SSc. Such research will help to inform future work to improve access to health information for people with SSc.

## Supplemental Material

sj-pdf-1-jso-10.1177_23971983251376428 – Supplemental material for Factors associated with eHealth literacy among people with systemic sclerosis: A Scleroderma Patient-centred Intervention Network (SPIN) Cohort cross-sectional study Supplemental material, sj-pdf-1-jso-10.1177_23971983251376428 for Factors associated with eHealth literacy among people with systemic sclerosis: A Scleroderma Patient-centred Intervention Network (SPIN) Cohort cross-sectional study by Natalie Co, Claire Adams, Marie-Eve Carrier, Meira Golberg, Elsa-Lynn Nassar, Linda Kwakkenbos, Susan J Bartlett, Catherine Fortuné, Amy Gietzen, Geneviève Guillot, Amanda Lawrie-Jones, Vanessa L Malcarne, Maureen D Mayes, Michelle Richard, Luc Mouthon, Andrea Benedetti and Brett D Thombs in Journal of Scleroderma and Related Disorders
