# Supplementary material for: Factors associated with eHealth literacy among people with systemic sclerosis: A Scleroderma Patient-centred Intervention Network (SPIN) Cohort cross-sectional study
Source: J Scleroderma Relat Disord. 2025 Sep 29:23971983251376428. Online ahead of print. doi: 10.1177/23971983251376428 (PMC12479466; doi:10.1177/23971983251376428)
Supplement: sj-pdf-1-jso-10.1177_23971983251376428 – Supplemental material for Factors associated with eHealth literacy among people with systemic sclerosis: A Scleroderma Patient-centred Intervention Network (SPIN) Cohort cross-sectional study [file sj-pdf-1-jso-10.1177_23971983251376428.pdf]

APPENDICES

Appendix 1. SPIN Investigators

Appendix 2. Complete case linear regression analysis of sociodemographic and disease characteristic associations with eHealth literacy (N = 306)

Appendix 3. Bivariate association of education and eHealth literacy by country

Appendix 4. Linear regression analysis of sociodemographic and disease characteristic associations with eHealth literacy, excluding France (N = 217)

Appendix 1. SPIN Investigators

Karen Gottesman, National Scleroderma Foundation, Los Angeles, California, USA; Marie Hudson, Lady Davis Institute for Medical Research, Jewish General Hospital and Department of Medicine, McGill University, Montreal, Quebec, Canada; Laura K. Hummers, Johns Hopkins University School of Medicine, Baltimore, Maryland, USA; Maureen Sauvé, Scleroderma Society of Ontario and Scleroderma Canada, Hamilton, Ontario, Canada; James Stempel, Scleroderma Chicago, Chicago, Illinois, USA; Robyn K. Wojeck, Amgen Inc., Thousand Oaks, California, USA; Christian Agard, Centre Hospitalier Universitaire - Hôtel-Dieu de Nantes, Nantes, France; Laurent Alric, CHU Rangueil, Toulouse, France; Marc André, Centre Hospitalier Universitaire Gabriel-Montpied, Clermont-Ferrand, France; Floryan Beaslay, CHU La Réunion, Saint-Denis, La Réunion, France; Elana J. Bernstein, Columbia University, New York, New York, USA; Sabine Berthier, Centre Hospitalier Universitaire Dijon Bourgogne, Dijon, France; Lyne Bissonnette, Université de Sherbrooke, Sherbrooke, Quebec, Canada; Sophie Blaise, CHU Grenoble Alpes, Grenoble, France; Eva Bories, CHU Rangueil, Toulouse, France; Alessandra Bruns, Université de Sherbrooke, Sherbrooke, Quebec, Canada; Carlotta Cacciatore, Assistance Publique – Hôpitaux de Paris, Hôpital St-Louis, Paris, France; Patricia Carreira, Servicio de Reumatologia del Hospital 12 de Octubre, Madrid, Spain; Marion Casadevall, Assistance Publique - Hôpitaux de Paris, Hôpital Cochin, Paris, France; Benjamin Chaigne, Assistance Publique - Hôpitaux de Paris, Hôpital Cochin, Paris, France; Lorinda Chung, Stanford University, Stanford, California, USA; Benjamin Crichi, Assistance Publique - Hôpitaux de Paris, Hôpital St-Louis, Paris, France; Thylbert Deltombe, CHU La Réunion, Saint-Denis, La Réunion, France; Christopher P. Denton, Royal Free London Hospital, London, UK; Tannvir Desroche, CHU La Réunion, Saint-Denis, La Réunion, France; Robyn Domsic, University of Pittsburgh, Pittsburgh, Pennsylvania, USA; James V. Dunne, St. Paul's Hospital and University of British Columbia, Vancouver, British Columbia, Canada; Bertrand Dunogue, Assistance Publique - Hôpitaux de Paris, Hôpital Cochin, Paris, France; Regina Fare, Servicio de Reumatologia del Hospital 12 de Octubre, Madrid, Spain; Dominique Farge-Bancel, Assistance Publique - Hôpitaux de Paris, Hôpital St-Louis, Paris, France; Paul R. Fortin, CHU de Québec - Université Laval, Quebec, Quebec, Canada; Tracy Frech, Vanderbilt University, Nashville, Tennessee, USA; Loraine Gauzère, CHU La Réunion, Saint-Denis, La Réunion, France; Anne Gerber, CHU La Réunion, Saint-Denis, La Réunion, France; Jessica K. Gordon, Hospital for Special Surgery, New York City, New York, USA; Brigitte Granel-Rey, Université, and Assistance Publique - Hôpitaux de Marseille, Hôpital Nord, Marseille, France; Aurélien Guffroy, Les Hôpitaux Universitaires de Strasbourg, Nouvel Hôpital Civil, Strasbourg, France; Geneviève Gyger, Jewish General Hospital and McGill University, Montreal, Quebec, Canada; Eric Hachulla, Centre Hospitalier Régional Universitaire de Lille, Hôpital Claude Huriez, Lille, France; Daphna Harel, New York University, NewYork, New York, USA; Monique Hinchcliff, Yale School of Medicine, New Haven, Connecticut, USA; Sabrina Hoa, Centre hospitalier de l'Université de Montréal – CHUM, Montreal, Quebec, Canada; Michael Hugues, Salford Royal NHS Foundation Trust, Salford, UK; Alena Ikic, CHU de Québec - Université Laval, Quebec, Quebec; Sindhu R. Johnson, Toronto Scleroderma Program, Mount Sinai Hospital, Toronto Western Hospital, and University of Toronto, Toronto, Ontario, Canada; Nader Khalidi, McMaster University, Hamilton, Ontario, Canada; Kimberly S. Lakin, Hospital for Special

Surgery, New York City, New York, USA; Marc Lambert, Centre Hospitalier Régional Universitaire de Lille, Hôpital Claude Huriez, Lille, France; Maggie Larche, University of Calgary, Calgary, Alberta, Canada; David Launay, Centre Hospitalier Régional Universitaire de Lille, Hôpital Claude Huriez, Lille, France; Yvonne C. Lee, Northwestern University, Chicago, Illinois, USA; Paul Legendre, Centre Hospitalier du Mans, Le Mans, France; Catarina Leite, University of Minho, Braga, Portugal; Hélène Maillard, Centre Hospitalier Régional Universitaire de Lille, Hôpital Claude Huriez, Lille, France; Nancy Maltez, University of Ottawa, Ottawa, Ontario, Canada; Joanne Manning, Salford Royal NHS Foundation Trust, Salford, UK; Isabelle Marie, CHU Rouen, Hôpital de Bois-Guillaume, Rouen, France; Maria Martin Lopez, Servicio de Reumatologia del Hospital 12 de Octubre, Madrid, Spain; Thierry Martin, Les Hôpitaux Universitaires de Strasbourg, Nouvel Hôpital Civil, Strasbourg, France; Ariel Masetto, Université de Sherbrooke, Sherbrooke, Quebec, Canada; Arsène Mekinian, Assistance Publique - Hôpitaux de Paris, Hôpital St-Antoine, Paris, France; Sheila Melchor Díaz, Servicio de Reumatologia del Hospital 12 de Octubre, Madrid, Spain; Morgane Mourguet, CHU Rangueil, Toulouse, France; Christelle Nguyen, Université Paris Descartes, Université de Paris, Paris, France, and Assistance Publique - Hôpitaux de Paris, Paris, France; Karen Nielsen, Scleroderma Society of Ontario, Hamilton, Ontario, Canada; Mandana Nikpour, St Vincent’s Hospital and University of Melbourne, Melbourne, Victoria, Australia; Louis Olagne, Centre Hospitalier Universitaire Gabriel-Montpied, Clermont-Ferrand, France; Vincent Poindron, Les Hôpitaux Universitaires de Strasbourg, Nouvel Hôpital Civil, Strasbourg, France; Janet Pope, University of Western Ontario, London, Ontario, Canada; Susanna Proudman, Royal Adelaide Hospital and University of Adelaide, Adelaide, South Australia, Australia; Grégory Pugnet, CHU Rangueil, Toulouse, France; Loïc Raffray, CHU La Réunion, Saint-Denis, La Réunion, France; François Rannou, Université Paris Descartes, Université de Paris, Paris, France, and Assistance Publique - Hôpitaux de Paris, Paris, France; Alexis Régent, Assistance Publique - Hôpitaux de Paris, Hôpital Cochin, Paris, France; Frederic Renou, CHU La Réunion, Saint-Denis, La Réunion, France; Sébastien Rivière, Assistance Publique - Hôpitaux de Paris, Hôpital St-Antoine, Paris, France; David Robinson, University of Manitoba, Winnipeg, Manitoba, Canada; Esther Rodríguez Almazar, Servicio de Reumatologia del Hospital 12 de Octubre, Madrid, Spain; Tatiana Sofia Rodríguez-Reyna, Instituto Nacional de Ciencias Médicas y Nutrición Salvador Zubirán, Mexico City, Mexico; Sophie Roux, Université de Sherbrooke, Sherbrooke, Quebec, Canada; Perrine Smets, Centre Hospitalier Universitaire Gabriel-Montpied, Clermont-Ferrand, France; Vincent Sobanski, Centre Hospitalier Régional Universitaire de Lille, Hôpital Claude Huriez, Lille, France; Robert F. Spiera, Hospital for Special Surgery, New York City, New York, USA; Virginia Steen, Georgetown University, Washington, DC, USA; Evelyn Sutton, Dalhousie University, Halifax, Nova Scotia, Canada; Carter Thorne, Southlake Regional Health Centre, Newmarket, Ontario, Canada; Damien Vagner, CHU La Réunion, Saint-Denis, La Réunion, France; John Varga, University of Michigan, Ann Arbor, Michigan, USA; Pearce Wilcox, St. Paul's Hospital and University of British Columbia, Vancouver, British Columbia, Canada; Vanessa Cook, Jewish General Hospital, Montreal, Quebec, Canada; Cassidy Dal Santo, Jewish General Hospital, Montreal, Quebec; Monica D’Onofrio, Jewish General Hospital, Montreal, Quebec; Sophie Hu, Jewish General Hospital, Montreal, Quebec, Canada.

Appendix 2. Complete case linear regression analysis of sociodemographic and disease characteristic associations with eHealth literacy (N = 306)

	Bivariate (Unadjusted) Regression Coefficient (95% CI)	Multivariable (Adjusted) Regression Coefficient (95% CI)
**Sociodemographic Variables**		
Age (per 10 years)	-0.40 (-1.00, 0.20)	-0.60 (-1.20, 0.10)
Sex (reference = female)	-1.49 (-3.74, 0.76)	-0.83 (-3.20, 1.54)
Non-White (reference = White)	0.44 (-1.37, 2.25)	0.36 (-1.57, 2.30)
Education (per 5 years)	0.15 (-0.60, 0.85)	-0.15 (-0.90, 0.60)
Single, divorced/separated, or widowed (reference = married or living as married)	0.43 (-1.14, 2.00)	0.13 (-1.52, 1.79)
Country (reference = United States)		
Canada	**-2.08 (-4.01, -0.15)**	-1.76 (-3.86, 0.33)
United Kingdom	-2.23 (-4.88, 0.41)	-1.39 (-4.73, 1.95)
France	**-3.87 (-5.70, -2.05)**	**-4.01 (-6.05, -1.98)**
Australia, Mexico, or Spain	**-5.05 (-9.31, -0.78)**	**-5.62 (-10.00, -1.23)**
Location (reference = city)		
Village or town	-0.86 (-2.74, 1.03)	-1.19 (-3.30, 0.91)
Suburb	0.36 (-1.44, 2.16)	-0.90 (-2.82, 1.03)
Rural	-0.15 (-2.61, 2.31)	-0.78 (-3.32, 1.76)
Years since first non-Raynaud's symptom (years)	0.00 (-0.08, 0.07)	-0.02 (-0.10, 0.06)

Diffuse subtype (reference = limited or sine)	0.28 (-1.19, 1.76)	-0.32 (-1.88, 1.24)

CI = confidence interval

Adjusted $R^2 = 0.03$. Statistically significant coefficients are shown in bold.

Appendix 3. Bivariate associations of education level (per 5 years) and eHealth literacy by country

	Bivariate (unadjusted) Regression Coefficient (95% CI)
Canada	1.00 (-1.20, 3.20)
United States	1.45 (-0.40, 3.30)
United Kingdom	1.40 (-1.80, 4.60)
France	-0.35 (-1.30, 0.55)

Appendix 4. Linear regression analysis of sociodemographic and disease characteristic associations with eHealth literacy, excluding France (N = 217)

	Bivariate (Unadjusted) Regression Coefficient (95% CI)	Multivariable (Adjusted) Regression Coefficient (95% CI)
**Sociodemographic variables**		
Age (per 10 years)	-0.40 (-1.20, 0.30)	-0.30 (-1.10, 0.50)
Sex (reference = female)	0.02 (-2.79, 2.82)	-0.40 (-3.26, 2.46)
Non-White (reference = White)	1.23 (-1.06, 3.53)	1.51 (-0.91, 3.94)
Education (per 5 years)	1.15 (-0.10, 2.40)	1.10 (-0.20, 2.40)
Single, divorced/separated, or widowed (reference = married or living as married)	-1.07 (-2.90, 0.77)	-1.43 (-3.37, 0.50)
Country (reference = United States)		
Canada	**-2.08 (-3.93, -0.23)**	-1.68 (-3.66, 0.30)
United Kingdom	-2.23 (-4.78, 0.31)	-1.83 (-4.71, 1.05)
Australia, Mexico, or Spain	**-5.05 (-9.14, -0.95)**	**-5.72 (-9.94, -1.49)**
Location (reference = city)		
Village or town	-0.02 (-2.34, 2.30)	-0.10 (-2.64, 2.44)
Suburb	0.58 (-1.51, 2.66)	-0.10 (-2.25, 2.05)
Rural	0.22 (-2.74, 3.17)	-0.51 (-3.52, 2.50)
Years since first non-Raynaud's symptom (years)	-0.06 (-0.15, 0.02)	-0.05 (-0.14, 0.04)

Diffuse subtype (reference = limited or sine)	-0.14 (-1.86, 1.59)	-0.78 (-2.57, 1.02)

CI = confidence interval
Adjusted R^2 = 0.03. Statistically significant coefficients are shown in bold.
